# Transfer RNA-derived small RNAs (tsRNAs): A rising star in liquid biopsy

**DOI:** 10.1016/j.gendis.2025.101608

**Published:** 2025-03-19

**Authors:** Qinglin Wang, Zehao Pan, Si Liang, Yuanjian Shi, Gaochao Dong, Lin Xu, Qixing Mao, Feng Jiang

**Affiliations:** a Department of Thoracic Surgery, Jiangsu Cancer Hospital, Jiangsu Institute of Cancer Research, Nanjing Medical University Affiliated Cancer Hospital, Nanjing, Jiangsu 210009, China; b Jiangsu Key Laboratory of Molecular and Translational Cancer Research, Nanjing Medical University Affiliated Cancer Hospital, Cancer Institute of Jiangsu Province, Nanjing, Jiangsu 210009, China

**Keywords:** Biomarker, Cancer, Diagnosis, Liquid biopsy, tsRNA

## Abstract

Liquid biopsy has emerged as a valuable clinical tool due to its non-invasive nature and real-time molecular profiling capabilities. Transfer RNA-derived small RNAs (tsRNAs) are a group of small non-coding RNAs generated from mature tRNAs or tRNA precursors. Recently, increasing studies have reported tsRNAs' potential to serve as promising biomarkers in various diseases, especially cancers. Notably, tsRNAs can be detected in various kinds of body fluids, such as seminal fluid plasma, blood plasma, saliva, and urine, and have been demonstrated to exist stably in body fluids. In this mini-review, we will summarize the recent discoveries on the role of tsRNAs in body fluids as biomarkers, hoping to provide new insights for disease diagnosis and therapeutic strategies.

## Introduction

Liquid biopsy has emerged as a valuable tool in clinical practice owing to its non-invasive nature and real-time molecular profiling capabilities. Different body fluids have been used for liquid biopsy, with blood being commonly regarded as the primary source. Nonetheless, other clinical samples such as saliva, sperm, pleural effusion, and urine have also been utilized.^1^, ^2^, ^3^, ^4^ Nevertheless, noninvasive samples, such as urine and saliva, despite potentially containing fewer biomarkers than blood, can still provide valuable insights into disease localization. For example, urine biomarkers may be associated with kidney or bladder conditions.^5^ Apart from potential candidates of liquid biopsy, such as circulating tumor cells and circulating tumor DNA, increasing numbers of studies reported that transfer RNA-derived small RNAs (tsRNAs) have the potential to be promising biomarkers in various diseases, especially in cancers.

Transfer RNAs (tRNAs) are essential for protein translation, as they recognize messenger RNA (mRNA) codons and transfer specific amino acids.^6^ Besides their canonical roles, tRNAs can generate a group of 18–40 nt small RNA fragments named tsRNAs, including tRNA-derived fragments (tRFs) and tRNA halves (tiRNAs). Since their discovery in the 1970s,^7^ tsRNAs were firstly considered as randomly degraded fragments of tRNA. Researchers later discovered that tsRNAs are generated from mature or precursor tRNAs through specific cleavage by enzymes such as angiogenin and Dicer.^8^^,^^9^ Although tsRNAs were ignored in the last century, with the advent of high-throughput RNA sequencing, tsRNAs were found to be evolutionarily conserved and structurally dependent and have organ-specific abundance, attracting many researchers' attention. In the past decades, increasing numbers of studies demonstrated that tsRNAs were dysregulated in various diseases, including neurodegenerative and metabolic disorders, viral infections, and cancers.^10^, ^11^, ^12^ Besides, tsRNAs exert biological effects through mechanisms such as protein interaction, transcription regulation, and translation regulation.^13^ Notably, tsRNAs can be detected in various kinds of body fluids, such as seminal fluid plasma, blood plasma, saliva, and urine,^14^ and were demonstrated to exist stably in body fluids.^15^

In this mini-review, we will summarize the recent discoveries of the role of tsRNAs as biomarkers, hoping to provide new insights for disease diagnosis and therapeutic strategies.

### Biogenesis and classification of tsRNAs

**Figure 1:**
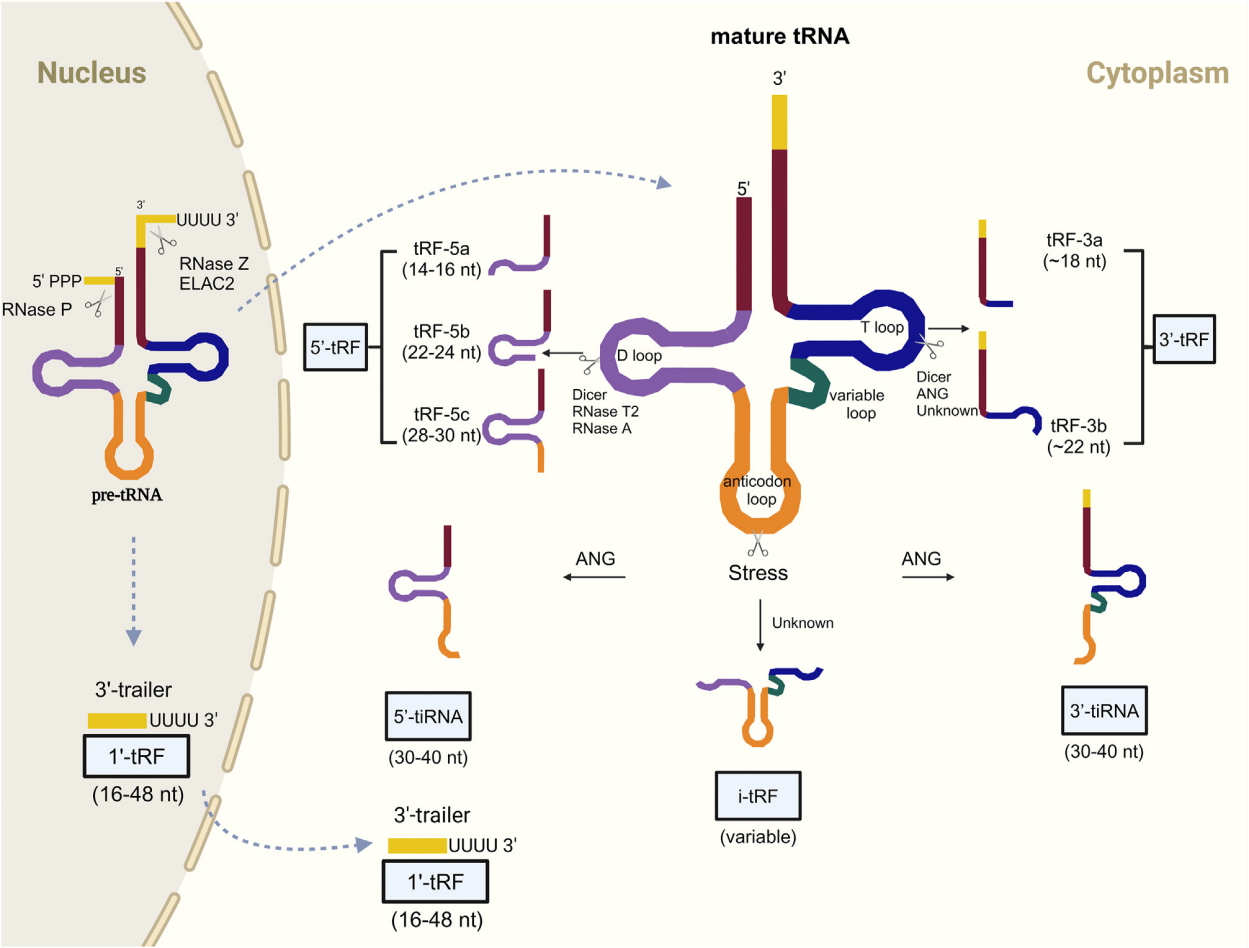
The classification of tsRNAs. tsRNAs are generated from mature tRNAs and pre-tRNAs. Depending on the cleavage site, tsRNAs have been classified into several types. The functions and biogenesis mechanisms of different types of tsRNAs remain poorly understood. The important modifications and modifying enzymes are shown.

tRNAs are transcribed by RNA polymerase III into precursor tRNAs in the nucleus. The maturation of pre-tRNA involves cleavage of the 5' leader sequence by RNase P and the 3' tail sequence by RNase Z. Subsequently, with the assistance of tRNA nucleotidyltransferase, a trinucleotide CCA is added to the 3' end. tsRNAs are generated from both mature tRNA and pre-tRNA through specific nuclease splicing. Based on the length and the cleavage site of tRNA, tsRNAs can be classified into three major groups: 5'tsRNAs, 3'tsRNAs, and other tsRNAs (Fig. 1).

#### 5'tsRNAs

The first group, known as 5'tsRNAs, originates from the 5' end of mature tRNAs to the region between D-loop and anticodon loop. Distinctions in the cleavage location allow for the classification of 5'tsRNAs into two subdivisions: 5'tRNA-derived fragments (5'tRFs) and 5'tRNA halves (5'tiRNAs). Cleaved by Dicer, RNase T2, or RNase A in the D-loop or stem region of mature tRNA, tRF-5a, tRF-5b, and tRF-5c were generated in the length of 14–16 nt, 22–24 nt, and 28–30 nt, respectively.^16^, ^17^, ^18^ Various stress conditions, such as oxidative stress, prompt tRNA fragmentation at the anticodon region through the influence of angiogenin, leading to the formation of 5'tiRNAs (30–40 nt).^19^, ^20^, ^21^, ^22^ These 5'tiRNAs, also referred to as 5'tRNA halves, possess a length nearly half that of mature tRNAs. Notably, apart from stress-mediated production, a subset of 5'tiRNAs has been reported to be regulated by sex hormones, highlighting the need for further investigation into their biogenesis mechanisms.^23^

#### 3'tsRNAs

The second group, known as 3'tsRNAs, originates from the 3' end of mature tRNAs, encompassing the region spanning from approximately the T loop to the 3' end (containing the CCA tail) of the tRNA molecule. Dicer, angiogenin, or other ribonucleases cleave on the TψC loop, resulting in the formation of two categories of 3'tRNA-derived fragments (3'tRFs): tRF-3a (∼18 nt) and tRF-3b (∼22 nt), with the length ranging from 13 to 22 nt^16^. Similar to 5'tiRNAs, 3'tRNA halves (3'tiRNAs) have 40–50 nt cleavage from mature tRNAs. 5'tsRNAs are mainly localized in the nucleus, whereas 3'tsRNAs are mostly localized in the cytoplasm.^24^

#### Other tsRNAs

Internal-tRFs (i-tRFs) and class I tRNA-derived fragments (tRF-1s) were classified as the third group, other tsRNAs, in this review.

i-tRFs, also named class II tRNA-derived fragments, originate from mature tRNAs. Due to the unique positioning of the break, this class of tsRNAs will encompass the corresponding tRNA anticodon loop. i-tRFs were firstly identified in breast cancer cell lines under hypoxic conditions.^25^ Based on the location of an i-tRF's 5' terminus along the mature tRNA, Telonis et al used to classify i-tRFs into six subtypes.^26^ The ribonucleases that cleave tRNAs to generate i-tRFs remain unknown.

tRF-1s are a group of 16–48 nt small RNAs derived from the 3' tails of precursor tRNAs. During tRNA maturation, tRF-1s are generated from the 3' end of precursor tRNAs through RNase Z cleaving in the nucleus, thus containing the' poly-U 'sequence. Interestingly, tRNA splicing commonly occurs in the nucleus, while tRF-1s are mostly located in the cytoplasm.^27^ The translocation and biological functions of tRF-1s were not sufficiently investigated. Besides, a previous study also reported that tRF-1s, such as tRF-1001, could be produced and regulated by elaC ribonuclease Z 2 (ELAC2) directly in the cytoplasm.^28^

Interestingly, the abundance of tiRNAs is asymmetric.^29^^,^^30^ The fate of 3'tiRNA and 5'tiRNA differs after tRNA cleavage. In some cases, both tRNA halves remain stable in the cell.^31^ Conversely, under stress-induced and some other conditions, the abundance of 5'tiRNA was significantly higher than 3'tiRNA.^30^, ^31^, ^32^, ^33^ These findings suggest that the majority of the remaining tRNA could be likely degraded by unknown enzymes. A recent study revealed that RNA helicases, such as DEAD-box helicase 3 X-linked (DDX3X), may participate in the degradation of 3'tiRNA after tRNA cleavage, causing asymmetry in the abundance of 5'tiRNA and 3'tiRNA.^34^ However, the fate of the remaining tRNA after the production of 3'tsRNA or 5'tsRNA remains poorly understood. Further investigations are warranted.

### Nomenclature of tsRNAs

Since the discovery of tsRNAs, tsRNAs were initially misclassified as microRNAs (miRNAs) or Piwi-interacting RNAs (piRNAs) owing to their similar size.^35^, ^36^, ^37^ Then, increasing research demonstrated that tsRNAs were a unique class of small RNAs derived from tRNAs and emerged as important players in various diseases. However, the nomenclature of tsRNAs is inconsistent and lacks standardized guidelines, leading to challenges in comparing findings among different studies and generating confusion.

Nowadays, although several tsRNA databases were established and widely used, these databases had created their own independent naming system and made inconvenience to researchers. For example, the same tsRNA sequence "TCCCCAGTACCTCCACCA" is assigned different names in various databases. In the tRFdb database, the assigned name for this tsRNA sequence is tRF-3019a, which is based on the time of its discovery.^24^ Meanwhile, it is designated as tDR-59:76-Ala-AGC-1 in the tDRmapper database, considering its specific location, parent tRNA, and mapped isodecoders.^38^ In Mintbase,^39^ it is identified as tRF-18-8*r*1546D2, while in the tsRFun database,^40^ it is referred to as tsRNA-Ala-3-0014, which is determined by applying a base-32 system in Mintbase and considering the parent tRNA, tsRNA type, and unique code in the tsRFun database, respectively.

The lack of standardization in the nomenclature of tsRNAs results in the presence of varied names for tsRNAs across different databases and research studies. Therefore, when conducting comparative analyses or referencing tsRNAs in their studies, researchers should take into consideration the specific naming conventions utilized by each resource to avoid any discrepancies. Although Holmes et al developed a new naming system and made efforts to propose a consistent one,^41^ the chaos surrounding tsRNA naming will continue for a long time and pose challenges to researchers.

### Clinical values of circulating tsRNAs in various diseases

**Figure 2:**
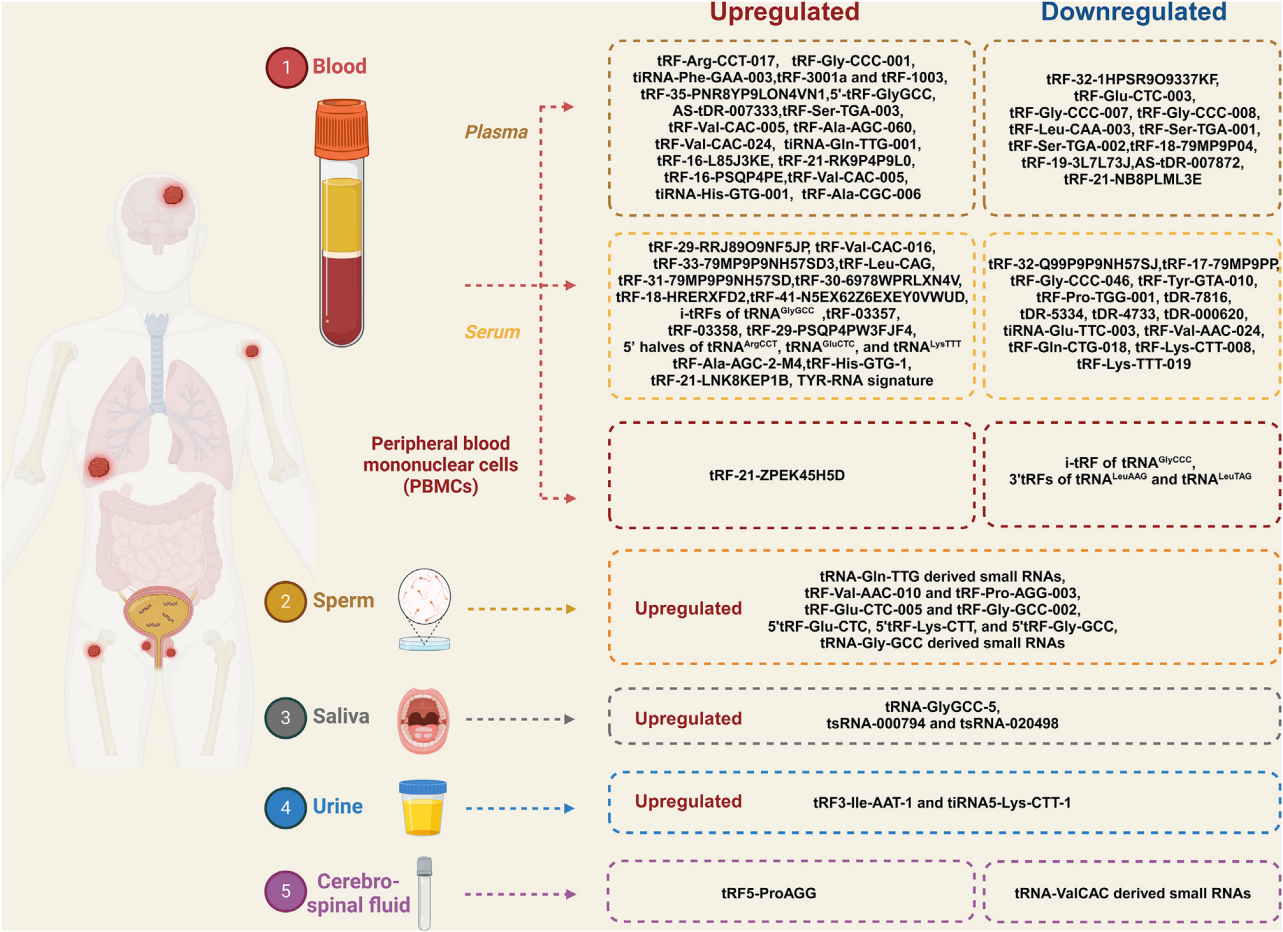
The reported circulating tsRNAs in body fluids, including blood, sperm, saliva, urine, and cerebrospinal fluid.

Since the identification of tsRNAs, increasing numbers of studies have revealed that tsRNAs play a regulatory role in various diseases, especially in cancers. The currently known mechanisms of tsRNA functions include mRNA silencing, nascent RNA silencing, regulation of translation, promoting ribosome biogenesis, *etc*. Meanwhile, it was found that tsRNAs can be detected in different kinds of body fluids.^14^ Interestingly, tsRNAs were demonstrated to be quite stable in body fluids. Nasim et al indicated that tsRNAs seem to be stabilized against nucleases by double-stranded RNA structures.^15^ Previous reviews have summarized relevant research on the regulation of cancer progression and its mechanisms. However, although a number of studies reported that tsRNAs could be promising biomarkers, few reviews focused on the diagnostic potential of tsRNAs in diseases. In this section, we will delineate a number of studies that focused on the diagnosis performance of tsRNAs according to the sample types (Fig. 2 and Table 1).

**Table 1:** The dysregulated tsRNAs in body fluids.

Diseases	Expression level	tsRNA	Sample type	Sample size	Clinical values	Reference
Breast cancer	Up-regulated	tRF-Arg-CCT-017, tRF-Gly-CCC-001, and tiRNA-Phe-GAA-003	Plasma	120 patients *vs*. 112 NCs	tsRNAs associated with overall survival and disease-free survival.	42
Breast cancer	Down-regulated	tRF-32-1HPSR9O9337KF	Plasma	47 patients *vs*. 23 NCs	Low expression significantly decreases in the blood of patients with epidermal growth factor receptor 2 (HER2)-positive breast cancer, reflecting tumor status.	43
Breast cancer	Up-regulated	tRF-3001a and tRF-1003	Plasma	19 patients *vs*. 19 NCs	tRF-3001a and tRF-1003 are up-regulated in the plasma of breast cancer patients and serve as better biomarkers than their miRNA counterparts in addition to miR-93–5p and miR-16–5p.	44
Breast cancer	Down-regulated	tRF-Glu-CTC-003, tRF-Gly-CCC-007, tRF-Gly-CCC-008, tRF-Leu-CAA-003, tRF-Ser-TGA-001, and tRF-Ser-TGA-002	Plasma	120 patients *vs*. 88 NCs	tsRNAs are significantly down-regulated in plasma samples and exosomes isolated from plasma in patients with early-stage breast cancer compared with NCs.	45
Gastric cancer	Down-regulated	tRF-18-79MP9P04	Plasma	86 patients *vs*. 37 NCs	Lower expression correlates with larger tumor size.	46
Gastric cancer	Down-regulated	tRF-19-3L7L73JD	Plasma	129 patients *vs*. 138 NCs	Lower expression correlates with larger tumor size.	47
Colorectal cancer	Up-regulated	tRF-35-PNR8YP9LON4VN1	Plasma	104 patients *vs*. 104 NCs	High expression correlates with pathological stage and metastasis.	48
Colorectal cancer	Up-regulated	5'-tRF-GlyGCC	Plasma	105 patients *vs*. 90 NCs	tsRNA is identified as a promising diagnostic biomarker for colorectal cancer diagnosis.	49
Lung cancer	Up-regulated	AS-tDR-007333	Plasma	29 patients *vs*. 45 NCs	High expression predicts poor survival of non-small cell lung cancer.	50
Lung cancer	Up-regulated	tRF-Ser-TGA-003, tRF-Val-CAC-005, tRF-Ala-AGC-060, tRF-Val-CAC-024, and tiRNA-Gln-TTG-001	Plasma	86 malignant *vs*. 14 benign	5'tsRNAs predict malignant pulmonary nodules and invasive malignant lesions.	51
Lung cancer	Down-regulated	AS-tDR-007872	Plasma	79 patients *vs*. 35 NCs	Plasma AS-tDR-007872 was identified as more sensitive in distinguishing stage III and IV than stage I-II non-small cell lung cancer patients.	52
Lung cancer	Up-regulated	tRF-16-L85J3KE, tRF-21-RK9P4P9L0 and tRF-16-PSQP4PE	Plasma	6 patients *vs*. 4 V	The area under the curve of the 3'tsRNAs' expression for diagnosing lung adenocarcinoma reaches 0.92.	53
Pathological cardiac hypertrophy	Down-regulated	tRF-21-NB8PLML3E	Plasma	35 patients *vs*. 25 NCs	The plasma tRF-21-NB8PLML3E is reported to be considered as a biomarker and offers early screening potential.	54
Nonalcoholic fatty liver disease	Up-regulated	tRF-Val-CAC-005, tiRNA-His-GTG-001, and tRF-Ala-CGC-006	Plasma	114 patients *vs*. 42 NCs	The expression level is associated with the activity score (calculated from 0 to 8) and fibrosis stage (scored from 0 to 4) of nonalcoholic fatty liver disease.	55
Breast cancer	Down-regulated	tRF-32-Q99P9P9NH57SJ	Serum	60 patients *vs*. 20 NCs	Lower expression in serum correlates with higher stage progression and lymph node metastasis.	56
Breast cancer	Down-regulated	tRF-17-79MP9PP	Serum	76 patients *vs*. 27 NCs	Lower expression in serum correlates with higher stage progression and lymph node metastasis.	57
Breast cancer	Down-regulated	tRF-Gly-CCC-046, tRF-Tyr-GTA-010 and tRF-Pro-TGG-001	Serum	214 patients *vs*. 113 NCs	tsRNAs are down-regulated in early-stage breast cancer.	58
Breast cancer	Down-regulated	tDR-7816, tDR-5334, and tDR-4733	Serum	45 patients *vs*. 22 NCs	tsRNAs are down-regulated in non-triple-negative breast cancer.	59
Breast cancer	Down-regulated	tDR-000620	Serum	44 patients *vs*. 28 NCs	Low tDR-000620 expression was demonstrated to be an independent adverse predictive factor for recurrence-free survival of triple-negative breast cancer.	60
Gastric cancer	Up-regulated	tRF-29-RRJ89O9NF5JP	Serum	124 patients *vs*. 19 NCs	The expression is positively associated with lymph node metastasis and tumor grade.	61
Gastric cancer	Up-regulated	tRF-Val-CAC-016	Serum	40 patients *vs*. 40 NCs	The expression is positively associated with lymph node metastasis and tumor grade.	62
Colorectal cancer	Up-regulated	tRF-33-79MP9P9NH57SD3	Serum	20 patients *vs*. 20 NCs	Overexpressed tRF-33-79MP9P9NH57SD3 is identified to positively correlate with lymph node and distant metastasis in colorectal cancer.	63
Lung cancer	Up-regulated	tRF-Leu-CAG	Serum	133 patients *vs*. 34 NCs	tRF-Leu-CAG is greatly up-regulated in stage III and stage IV cases and increases with stage progression.	64
Lung cancer	Up-regulated	tRF-31-79MP9P9NH57SD	Serum	96 patients *vs*. 96 NCs	Serum tRF-31-79MP9P9NH57SD expression is reported to be positively associated with clinical stage and the malignancy of lymph node of non-small cell lung cancer.	65
Pancreatic cancer	Up-regulated	tRF-30-6978WPRLXN4V, tRF-18-HRERXFD2	Serum	30 patients *vs*. 30 NCs	tRF-30-6978WPRLXN4V-high and tRF-18-HRERXFD2-high pancreatic cancer patients show significantly worse prognosis.	66
Pancreatic cancer	Up-regulated	tRF-41-N5EX62Z6EXEY0VWUD	Serum	110 patients *vs*. 100 NCs	High tRF-41-N5EX62Z6EXEY0VWUD expression is associated with advanced tumor stage and liver metastasis and predicts poor overall survival in pancreatic cancer.	67
Ovarian cancer	Up-regulated	i-tRFs of tRNAGlyGCC	Serum	64 high-grade serous, 14 low-grade serous, 9 mucinous, 6 endometrioid, 2 clear cell carcinomas, and 3 undifferentiated carcinomas	Then i-tRFs of tRNAGlyGCC are identified as being associated with advanced stages, suboptimal debulking, and early progression, and they predict poor overall survival of early ovarian cancer.	69
Ovarian cancer	Up-regulated	tRF-03357 and tRF-03358	Serum	23 patients *vs*. 18 NCs	tRF-03357 and tRF-03358 are significantly increased in high-grade serous ovarian cancer.	70
Bladder cancer	Up-regulated	tRF-29-PSQP4PW3FJF4	Serum	141 patients of Ta, T1 stage *vs*. 89 patients of T2-T4 stage	High expression of tRF-29-PSQP4PW3FJF4 is associated with aggressive tumor phenotype, early disease progression, and poor treatment outcome.	71
Renal cancer	Up-regulated	5’ halves of tRNA^ArgCCT^, tRNA^GluCTC^, and tRNA^LysTTT^	Serum	27 patients *vs*. 13 NCs	Lower expression levels of 5' halves of tRNA^ArgCCT^, tRNA^GluCTC^, and tRNA^LysTTT^ are demonstrated to be associated with adverse clinicopathological parameters.	72
Lupus nephritis	Up-regulated	tRF-Ala-AGC-2-M4	Serum	130 patients *vs*. 193 NCs	tRF-Ala-AGC-2-M4 is reported to have a good diagnostic value for lupus nephritis and is closely related to the severity of lupus nephritis.	74
Radiation exposure	Down-regulated	tiRNA-Glu-TTC-003, tRF-Val-AAC-024, tRF-Gln-CTG-018, tRF-Lys-CTT-008, and tRF-Lys-TTT-019	Serum	6 pre-exposure patients *vs*. 6 post-exposure patients	Circulating tsRNAs can serve as minimally invasive biomarkers for triage or dose assessment in exposure scenarios, with blood sample collection within 4 h.	75
Systemic lupus erythematosus	Up-regulated	tRF-His-GTG-1	Serum	52 patients *vs*. 86 NCs	The combination of tRF-His-GTG-1 and anti-dsDNA could serve as biomarkers for diagnosing systemic lupus erythematosus with a high area under the curve of 0.95.	76
Enthesitis-related arthritis	Up-regulated	tRF-21-LNK8KEP1B	Serum	30 patients *vs*. 31 NCs	tRF-21-LNK8KEP1B is substantially increased in the serum of enthesitis-related arthritis patients compared with that in healthy controls.	77
Lung cancer	Up-regulated	TYR-RNA signature	PBMCs	51 lung cancer patients *vs*. 25 NCs *vs*. 17 pulmonary tuberculosis patients	Distinguishing healthy control, lung cancer, and pulmonary tuberculosis patients	78
Chronic lymphocytic leukemia	Down-regulated	tRF-21-ZPEK45H5D	PBMCs	91 patients *vs*. 43 NCs	Overexpression of tRF-21-ZPEK45H5D is related to poor overall survival of chronic lymphocytic leukemia patients.	79
Chronic lymphocytic leukemia	Up-regulated	i-tRF of tRNAGlyCCC	PBMCs	90 patients *vs*. 43 NCs	Positive expression of i-tRF of tRNAGlyCCC reduced overall survival for chronic lymphocytic leukemia patients.	80
B-cell chronic lymphocytic leukemia	Up-regulated	3'tRFs from tRNALeuAAG and tRNALeuTAG	PBMCs	91 patients *vs*. 43 NCs	tsRNAs associate with poor prognosis in B-cell chronic lymphocytic leukemia, independently of classical prognostic factors.	81
Male infertility	Up-regulated	tRNA-Gln-TTG derived small RNAs	Sperm	21 high-quality patients *vs*. 64 low-quality patients	tsRNAs serve as potential diagnostic biomarkers and clinical targets for male infertility.	84
Non-obstructive azoospermia	Up-regulated	tRF-Val-AAC-010 and tRF-Pro-AGG-003	Sperm	23 patients *vs*. 12 NCs	tRF-Val-AAC-010 has been speculated to have the potential to serve as a non-invasive biomarker for predicting the presence of sperm in non-obstructive azoospermia testicular tissue.	85
Non-obstructive azoospermia	Up-regulated	tRF-Glu-CTC-005 and tRF-Gly-GCC-002	Sperm	18 patients *vs*. 12 NCs	tsRNAs serve as predictive factors for successful microdissection testicular sperm extraction in patients with non-obstructive azoospermia.	86
Assisted reproductive technology	Up-regulated	5'tRF-Glu-CTC, 5'tRF-Lys-CTT, and 5'tRF-Gly-GCC	Sperm	25 patients *vs*. 31 NCs	There is a potential association between increased seminal tRFs and unexplained male infertility.	87
Esophageal squamous cell carcinoma	Up-regulated	tRNA-GlyGCC-5	Saliva	237 patients *vs*. 137 NCs	tsRNA could play a potential role in diagnosis and prognosis and function as a preoperative biomarker to aid in the selection of patients who could potentially benefit from adjuvant therapy.	91
Response to *F. Nucleatum*	Up-regulated	tsRNA-000794 and tsRNA-020498	Saliva	–	tsRNAs play a potential role in the microbial–host interaction.	92
Lupus nephritis	Up-regulated	tRF3- Ile-AAT-1 and tiRNA5-Lys-CTT-1	Urinary Exosomes	54 patients *vs*. 39 NCs	tsRNAs serve as novel biomarkers for the diagnosis of lupus nephritis.	93
Fetal congenital heart disease during pregnancy	Up-regulated	tRF-58:74-Gly-GCC-1	Maternal Serum	41 patients *vs*. 54 NCs	tsRNA serves as a new potential biomarker for the accurate diagnosis of fetal congenital heart disease during pregnancy.	94
Alzheimer's disease	Down-regulated	tRF5-ProAGG	Cerebrospinal Fluid	24 patients *vs*. 17 NCs	tRF5-ProAGG showed potential as an Alzheimer's disease biomarker and may play a role in disease progression.	95
Progressive supranuclear palsy	Up-regulated	tRNA-ValCAC	Cerebrospinal Fluid	20 patients *vs*. 20 NCs	tsRNAs could be possible diagnostic biomarkers for progressive supranuclear palsy, and further work will help to expand this potential.	96

Note: PBMCs, peripheral blood mononuclear cells; NCs, normal controls.

#### tsRNAs in plasma

tsRNA is currently detectable and extensively studied in plasma and serum, particularly in the field of cancer research. By harnessing the stability and detectability of tsRNAs, we may be able to develop non-invasive diagnostic tests and monitor treatment response in a more efficient and convenient manner.

Numerous tsRNAs have been identified as potential biomarkers in the diagnosis and treatment of various types of cancer. For instance, in breast cancer, several circulating tsRNAs were found to be up-regulated in the plasma samples of breast cancer patients. Notably, the expression of circulating tRF-Arg-CCT-017 and tiRNA-Phe-GAA-003 correlated with overall survival and disease-free survival, suggesting their utility as biomarkers.^42^ Conversely, the level of tRF-32-1HPSR9O9337KF significantly decreased in the blood of patients with epidermal growth factor receptor 2 (HER2)-positive breast cancer, reflecting tumor status.^43^ In another study, up-regulated tRF-3001a and tRF-1003 in plasma samples showed superior biomarker potential compared with their miRNA counterparts, including miR-93–5p and miR-16–5p.^44^ Additionally, six tsRNAs were found to be significantly down-regulated in plasma samples and exosomes isolated from patients with early-stage breast cancer compared with normal controls.^45^ In gastric cancer, two tsRNAs, namely tRF-18-79MP9P04 and tRF-19-3L7L73JD, were identified to have low expression in plasma and were found to be correlated with larger tumor size.^46^^,^^47^ Furthermore, high expression of tRF-35-PNR8YP9LON4VN1 in plasma was demonstrated to correlate with the pathological stage and metastasis of colorectal cancer.^48^ The level of 5'-tRF-GlyGCC in plasma was also identified as a promising diagnostic biomarker in colorectal cancer diagnosis.^49^ In lung cancer, high expression of AS-tDR-007333 was identified as a predictor for poor survival in patients with non-small cell lung cancer.^50^ In our previous study, a nomogram was developed to predict malignant pulmonary nodules and invasive malignant lesions based on the levels of five circulating tsRNAs.^51^ Additionally, plasma AS-tDR-007872 was found to have the potential to distinguish stage III and IV non-small cell lung cancer patients compared with stage I-II.^52^ Another study reported that the expression levels of three tsRNAs achieved an area under the curve of 0.92 for diagnosing lung adenocarcinoma.^53^

In other diseases, plasma tsRNAs have also been explored for their applications. For instance, plasma tRF-21-NB8PLML3E was reported to have early screening potential in patients with pathological cardiac hypertrophy.^54^ In nonalcoholic fatty liver disease, three tsRNAs exhibited significant elevation in the plasma of the patients, and their expression levels correlated with the disease activity score and fibrosis stage.^55^ Overall, tsRNA holds promise as a candidate for biomarker discovery and diagnostic applications.

#### tsRNAs in serum

The main difference between plasma and serum is that plasma is obtained by collecting blood samples with an anticoagulant, while serum is obtained after allowing the blood sample to clot and removing the clot and blood cells. Plasma contains clotting factors, while serum does not. In this section, we will summarize the research on serum tsRNA.

The studies on tsRNA in serum have contributed significantly to the field. For example, in breast cancer, two tsRNAs in serum showed lower expression levels that correlated with higher stage and metastasis.^56^^,^^57^ Besides, down-regulated tRF-Gly-CCC-046, tRF-Tyr-GTA-010, and tRF-Pro-TGG-001 were indicated in early-stage breast cancer^58^ while tDR-7816, tDR-5334, and tDR-4733 were down-regulated in non-triple-negative breast cancer.^59^ The independent prognostic significance of reduced tDR-000620 expression was identified in triple-negative breast cancer, indicating its association with a higher risk of recurrence and shorter recurrence-free survival.^60^ In gastric cancer, the expression of two circulating tsRNAs in serum were positively associated with tumor progression.^61^^,^^62^ Further, overexpressed tRF-33-79MP9P9NH57SD3 was identified to positively correlate with metastasis in colorectal cancer.^63^ In lung cancer, tRF-Leu-CAG was associated with stage III and stage IV cases and increased with progression.^64^ Further, serum tRF-31-79MP9P9NH57SD expression was positively associated with tumor progression in non-small cell lung cancer.^65^ In the studies of pancreatic cancer, Jin et al identified that high expression of two tsRNAs in pancreatic cancer patients was significantly associated with a worse prognosis.^66^ Importantly, this study demonstrated the correlation between the abundance of circulating tsRNA and the abundance of tsRNA in tumors, indicating that circulating tsRNA could originate from tumors. Another study reported that one tsRNA expression was associated with tumor progression and poor overall survival in pancreatic cancer.^67^ The field of ovarian cancer research also involves serum tsRNA. Eric et al reported the presence of circulating tsRNA in ovarian tumor patients' serum.^68^ i-tRFs of tRNAGlyGCC were associated with advanced stages, suboptimal debulking, early progression, and poor overall survival in early ovarian cancer.^69^ tRF-03357 and tRF-03358 were also found to be significantly up-regulated in high-grade serous ovarian cancer.^70^ In bladder cancer, high expression of tRF-29-PSQP4PW3FJF4 was associated with an aggressive tumor phenotype, early disease progression, and poor treatment outcome.^71^ In renal cancer, lower expression levels of 5' halves of tRNA^ArgCCT^, tRNA^GluCTC^, and tRNA^LysTTT^ were demonstrated to be associated with adverse clinicopathological parameters.^72^

The application of serum tsRNA has also been explored in various other diseases by numerous research studies. Zhang et al have identified a class of tsRNAs that abundantly and conservatively exist across a wide range of vertebrate species and demonstrate their sensitive response to body infection in mouse, monkey, and human being, revealing the potential link between tsRNAs and infection.^73^ In terms of disease biomarkers, Zhang et al have identified that tRF-Ala-AGC-2-M4 has good diagnostic value for lupus nephritis and is closely related to the severity of lupus nephritis.^74^ Circulating tsRNAs were also identified as potential biomarkers for radiation exposure in another study.^75^ After exposure to carbon ions, protons, or X-rays, 5'tsRNAs showed a significant decrease in serum levels. These findings suggest that circulating tsRNAs can serve as non-invasive biomarkers for triage or dose assessment in exposure scenarios, with blood samples collected within 4 h. In systemic lupus erythematosus, the combination of tRF-His-GTG-1 and anti-dsDNA could be used for diagnosing, with a high area under the curve of 0.95^76^. In enthesitis-related arthritis, tRF-21-LNK8KEP1B was substantially up-regulated in the serum of the patients, with an area under the curve of 0.7849.^77^

#### tsRNAs in peripheral blood mononuclear cells

Peripheral blood mononuclear cells are a diverse group of immune cells that circulate in the blood, including lymphocytes, monocytes, and dendritic cells, which play crucial roles in immune responses against pathogens and tumor cells. The major studies of tsRNAs in peripheral blood mononuclear cells were conducted in the field of lung cancer and leukemia.

In the field of lung cancer screening, Gu et al established a TYR-RNA signature based on 9 tsRNAs, 8 rsRNAs, and 8 ysRNAs and achieved a robust performance in distinguishing healthy controls, lung cancer patients, and pulmonary tuberculosis patients.^78^ In the field of chronic lymphocytic leukemia, overexpression of tRF-21-ZPEK45H5D is associated with poorer overall survival.^79^ Additionally, positive expression of i-tRF of tRNA^GlyCCC^ has been found to reduce overall survival in patients with chronic lymphocytic leukemia.^80^ Furthermore, a 3'tRNA-derived fragment generated by tRNA^LeuAAG^ and tRNA^LeuTAG^ has been linked to a worse prognosis in B-cell chronic lymphocytic leukemia, independent of traditional prognostic factors.^81^ More research is required to explore the potential applications of tsRNAs in peripheral blood mononuclear cells.

#### tsRNAs in sperm

tsRNAs were firstly identified as highly accumulation in mouse sperm during spermatogenesis.^82^ Recently, researchers have indicated that tsRNAs present in sperm may have potential applications as biomarkers for various diseases. Sharma et al conducted a study on the formation and role of tsRNAs during sperm maturation and fertilization, highlighting their relevance in reproductive biology and assisted reproductive technologies.^83^ In recent studies, aberrant expression levels of tsRNAs in sperm have been associated with male infertility and nonobstructive azoospermia.

Certain small RNAs derived from tRNA^GlnTTG^ in human sperm have been proposed as possible diagnostic biomarkers and therapeutic targets for male infertility.^84^ In cases of non-obstructive azoospermia, two tsRNAs have been identified as biomarkers for diagnosing this condition, with tRF-Val-AAC-010 even being indicated to have the potential to predict the presence of sperm in testicular tissue affected by non-obstructive azoospermia.^85^ Furthermore, Zhang et al demonstrated that two circulatory exosomal tsRNAs could be used for predicting successful microdissection testicular sperm extraction in patients with non-obstructive azoospermia.^86^ In the context of assisted reproductive technology, elevated levels of 3'tsRNAs were observed in seminal samples from individuals with repeated failed intracytoplasmic sperm injection cycles, indicating a potential link between increased seminal tsRNAs and unexplained male infertility.^87^ Additionally, tsRNAs in sperm have been found to contribute to metabolic disorders induced by paternal inflammation in offspring.^88^^,^^89^ Parental exposure to endocrine-disrupting chemicals was proved to cause changes in sperm RNA (miRNAs and tsRNAs), indicating the potential application of tsRNAs as sperm biomarkers.^90^

#### tsRNAs in saliva

tsRNA can stably exist in various body fluids and could be easily detected, including saliva.^14^ tsRNA in saliva has gradually attracted the attention of researchers recently.

tsRNA in saliva was proposed as a potential biomarkers for esophageal squamous cell carcinoma. Li et al developed a cancer-enriched tsRNA signature and identified salivary exosome tsRNA generated from tRNA-GlyGCC-5 as a non-invasive biomarker for human esophageal squamous cell carcinoma.^91^ The potential utility of the tsRNA-based signature extends beyond diagnosis and prognosis, as it may also function as a preoperative biomarker to aid in the selection of patients who could potentially benefit from adjuvant therapy. Additionally, another study^92^ demonstrated the presence of diverse tsRNAs in human salivary small RNAs and identified two tsRNAs that are released by oral keratinocyte cells in response to *F. nucleatum*. These tsRNAs inhibited the growth of *F. nucleatum* but not *Streptococcus mitis*, indicating their potential role in the interaction between microorganisms and the host.

#### tsRNAs in other body fluids

Apart from the body fluids mentioned above, several studies also focused on the tsRNAs in urine, maternal serum, and cerebrospinal fluid.

In systemic lupus erythematosus, lupus nephritis (LN) is a severe organ manifestation. Chen et al found that two tsRNA signatures in urinary exosomes can be used for diagnosing lupus nephritis.^93^ Elevated levels of the two tsRNAs were observed in urinary exosomes from lupus nephritis patients compared with those without lupus nephritis and healthy controls. The area under the curve for distinguishing lupus nephritis from systemic lupus erythematosus patients without lupus nephritis and healthy controls for the two tsRNAs was 0.777 and 0.715, respectively. In fetal congenital heart disease, maternal serum was found to contain one tsRNA, which could serve as a potential biomarker for accurate prenatal diagnosis.^94^ In Alzheimer's disease, tRF5-ProAGG was identified as down-regulated in both cerebrospinal fluid and serum and may have potential as a biomarker for Alzheimer's disease and disease progression.^95^ Furthermore, the up-regulated expression of tRNA-ValCAC in cerebrospinal fluid was reported as a possible diagnostic biomarker for progressive supranuclear palsy.^96^

In summary, the above studies suggest that tsRNAs in body fluids may have the potential to be used as biomarkers for various diseases. However, further research is needed to fully understand their diagnostic and prognostic value in clinical settings.

## Conclusions and prospectives

tsRNAs have gained widespread interest due to their stable presence and easy detectability in various body fluids. This stability makes them an attractive candidate for biomarker discovery and diagnostic applications. Additionally, the presence of tsRNAs in various body fluids holds great potential for non-invasive disease diagnosis and monitoring. The ease of detecting tsRNAs opens up opportunities for developing novel diagnostic tools and therapeutic strategies.

Although similar in length, studies have demonstrated the different expression profiles between tsRNAs and miRNAs in body fluids.^14^ Shaharbhanu et al found that tRF-3001a and tRF-1003 are superior biomarkers compared with their corresponding miRNAs.^44^ It is crucial to employ meticulously designed experiments and critically interpret data to differentiate tsRNAs from miRNAs in tsRNA research. In addition, recent studies have provided evidence suggesting that the production of tsRNAs is controlled by modifications of tRNAs. These modifications directly impact the formation and roles of tsRNAs. For instance, multiple investigations have shown that the methylation of tRNAs at 5-methylcytosine sites, mediated by the enzymes Dnmt2 and NSun2, safeguards tRNAs from being cleaved into 5′tsRNAs and decreases translation speed.^97^^,^^98^ The modifications of tRNAs and biogenesis of tsRNAs remain an attractive field.

Although the application of tsRNAs as biomarkers holds clinical value and attractive prospects, there are still several challenges that need to be addressed in utilizing tsRNAs as disease markers. For example, despite the demonstrated effectiveness of tsRNAs as satisfactory biomarkers in various diseases, the lack of a unified standard remains a challenge. Furthermore, extracellular tsRNAs were reported to exist in double-stranded RNA structures to stabilize against nucleases,^15^ partly explaining the reason for the stable existence of tsRNA in body fluids. However, the structure of tsRNA in body fluids has not been fully studied and remains an attractive field. Meanwhile, although many studies have focused on tsRNAs in blood, the potential and clinical values of tsRNAs in other body fluids should not be ignored. Additionally, the lack of standardization in the nomenclature of tsRNAs results in the presence of varied names for tsRNAs across different databases and research studies. The chaos surrounding tsRNA naming will continue for a long time and pose challenges to researchers.

Nevertheless, the quality control of body fluid samples and the detection or quantification of tsRNAs could also pose challenges for researchers. On the one hand, as various body fluids could be used for tsRNA detection, comprehensive liquid sample quality control is essential for future research; on the other hand, the implementation of optimized sequencing methods are essential for identification and quantification of tsRNA candidates in various body fluids, such as PANDORA-seq^99^ and Cap-Clip acid pyrophosphatase, PNK, and CPA-seq.^100^ As a member of small RNA classes, choosing sRNA annotation software aiming to analyze tsRNAs in addition to other canonical sRNAs, such as SPORTS,^101^ is also a crucial step in tsRNA research. Furthermore, studies reported that the abundance of tsRNA could be tissue-specific and associated with aging.^102^, ^103^, ^104^, ^105^ Hence, it is imperative to consider whether the research outcomes on tsRNAs could be potentially influenced by age disparities when a cohort is engaged in the study. The levels of tsRNAs appear to be influenced by aging. When studying tsRNA biomarkers or constructing a predictive model, should the age of the cohort be controlled? Additionally, as the abundance of tsRNA could be tissue-specific, the comparability of tsRNA levels across various bodily fluids also warrants attention. These factors unequivocally warrant thoughtful consideration in any research paradigm.

In summary, the stable presence and detectability of tsRNAs in various body fluids make them an intriguing area of research. Investigating their therapeutic and diagnostic potential could result in significant advancements in personalized medicine and disease management. Future studies are required to improve our understanding and applications of tsRNAs in diseases.

## CRediT authorship contribution statement

**Qinglin Wang:** Writing – original draft. **Zehao Pan:** Writing – original draft. **Si Liang:** Writing – original draft. **Yuanjian Shi:** Writing – original draft. **Gaochao Dong:** Writing – review & editing. **Lin Xu:** Writing – review & editing. **Qixing Mao:** Writing – review & editing. **Feng Jiang:** Writing – review & editing.

## Funding

This study was supported by the grants from the 10.13039/100014717National Natural Science Foundation of China (No. 82073211, 82002434, 82003106); The Project of Invigorating Health Care through Science, Technology and Education, Jiangsu Provincial Medical Innovation Team (China) (No. CXTDA2017002); The Project of Invigorating Health Care through Science, Technology and Education, Jiangsu Provincial Medical Outstanding Talent (China) (No. JCRCA2016001); and Young Talents Program of Jiangsu Cancer Hospital (China) (No. 23).

## Conflict of interests

The authors declared no competing interests.
